# Effectiveness of Facebook Groups and Pages on Participant Recruitment Into a Randomized Controlled Trial During the COVID-19 Pandemic: Descriptive Study

**DOI:** 10.2196/46190

**Published:** 2023-10-17

**Authors:** Kirstie H T W Wong, Wallis C Y Lau, Kenneth K C Man, Andrea Bilbow, Patrick Ip, Li Wei

**Affiliations:** 1 Research Department of Practice and Policy University College London London United Kingdom; 2 Department of Paediatrics and Adolescent Medicine Li Ka Shing Faculty of Medicine University of Hong Kong Hong Kong China (Hong Kong); 3 Centre for Safe Medication Practice and Research, Department of Pharmacology and Pharmacy Li Ka Shing Faculty of Medicine University of Hong Kong Hong Kong China (Hong Kong); 4 Laboratory of Data Discovery for Health Hong Kong Science Park Hong Kong China (Hong Kong); 5 National Attention Deficit Disorder Information and Support Services London United Kingdom

**Keywords:** 1-2-3 Magic, ADHD, attention deficit/hyperactivity disorder, behavioral parenting training, BPT, clinical trial, COVID-19, Facebook group, Facebook page, Facebook, pediatric, randomized controlled trial, recruitment, social media, youth, Zoom

## Abstract

**Background:**

In response to the unprecedented challenges posed by the COVID-19 pandemic, conventional recruitment approaches were halted, causing the suspension of numerous clinical trials. Previously, Facebook (Meta Platforms, Inc) has emerged as a promising tool for augmenting participant recruitment. While previous research has explored the use of Facebook for surveys and qualitative studies, its potential for recruiting participants into randomized controlled trials (RCTs) remains underexplored.

**Objective:**

This study aimed to comprehensively examine the effectiveness of using Facebook groups and pages to facilitate participant recruitment during the COVID-19 pandemic for an RCT on the effectiveness of a remote parenting program, 1-2-3 Magic, in families who have children with attention-deficit/hyperactivity disorder (ADHD) in the United Kingdom.

**Methods:**

We disseminated 5 Facebook posts with an attached digital flyer across 4 prominent ADHD UK support groups and pages run by the National Attention Deficit Disorder Information and Support Services, reaching an audience of around 16,000 individuals over 2 months (January 7 to March 4, 2022). Eligibility criteria mandated participants to be parents or caregivers of a child with diagnosed ADHD aged 12 years or younger, be residing in the United Kingdom, have access to stable internet, and have a device with the Zoom (Zoom Video Communications) app. Participants were required to have never attended 1-2-3 Magic training previously. Prospective participants expressed their interest through Microsoft Forms (Microsoft Corporation). The trial aimed to recruit 84 parents. It is important to note that the term “parent” or “caregiver” in the RCT and in this study within a trial refers to anybody who has legal responsibility for the child.

**Results:**

Overall, 478 individuals registered their interest through Microsoft Forms within the stipulated 2-month window. After the eligibility check, 135 participants were contacted for a baseline meeting through Zoom. The first 84 participants who attended a baseline meeting and returned a completed consent form were enrolled. Subsequently, another 16 participants were added, resulting in a final sample of 100 participants. This recruitment strategy incurred negligible expenses and demanded minimal human resources. The approach yielded favorable outcomes by efficiently attracting eligible participants in a condensed time frame, transcending geographical barriers throughout the United Kingdom, which would have been tedious to achieve through traditional recruitment methods.

**Conclusions:**

Our experience demonstrated that digital flyers posted in the targeted Facebook groups were a cost-effective and quick method for recruiting for an RCT, which opened during the COVID-19 pandemic when lockdown restrictions were in place in the United Kingdom. Trialists should consider this low-cost recruitment intervention for trials going forward, and in the case of a global pandemic, this novel recruitment method enabled the trial to continue where many have failed.

**Trial Registration:**

International Standard Randomized Controlled Trial Number (ISRCTN) 15281572; https://www.isrctn.com/ISRCTN15281572

## Introduction

With the rise of the COVID-19 pandemic, which swept over almost the entire world, our society had to adapt to remote working and using the internet and web-based resources in every sphere of life [1]. Many planned clinical trials were put on hold due to the pandemic, and conventional methods of recruitment were not encouraged as face-to-face contact had to be limited [2-5]. Using social media to enhance recruitment and promotion for clinical trials has been suggested; however, the sole use of social media for participant recruitment in clinical trials has been underexplored [6,7].

Facebook (Meta Platforms, Inc) is one of the most commonly used social media platforms for clinical trial recruitment. A meta-analysis looking into the use of Facebook for recruiting participants was conducted with 6 studies [8]. However, they only explored paid advertising, the use of the Facebook search bar, and creating Facebook pages. A total of 4 out of 6 studies also used traditional recruitment and snowballing methods alongside Facebook. Since the publication of the meta-analysis, further studies have explored paid Facebook advertising for their studies [9-16]. A previous study [17] also used established Facebook pages and groups to recruit for a qualitative study; however, they deemed the method not appropriate for obtaining larger samples needed for quantitative research. They came to this conclusion because they did not deem it possible to obtain enough participants needed for a clinical trial; however, this conclusion should not be generalized to all cases. Therefore, the effectiveness of using established Facebook groups and pages for recruiting for quantitative studies remains unclear.

During the COVID-19 pandemic, we conducted a quantitative randomized controlled trial (RCT) to evaluate the effectiveness of 1-2-3 Magic when delivered remotely. 1-2-3 Magic is a parenting program designed for parents of children aged between 2 and 12 years with attention-deficit/hyperactivity disorder (ADHD). It aims to aid parents with neurodivergent-specific tools for healthy parenting, communication, and bonding and to create a sphere for parents with similar situations and struggles to support one another, under the supervision of a trained practitioner. Due to the COVID-19 pandemic, we could not use traditional methods; therefore, we used established Facebook groups and pages to recruit for our quantitative RCT. Thus, the aim of this paper was to explore the effectiveness of using established Facebook groups and pages to recruit participants for a quantitative RCT.

## Methods

### Overview of the 1-2-3 Magic RCT

1-2-3 Magic is a parenting behavioral program designed by American psychologist Dr Thomas Phelan for children with ADHD [18]. The RCT aimed to study the effectiveness of 1-2-3 Magic on the web in families of children with ADHD in a UK cohort and is the first trial on 1-2-3 Magic to be conducted remotely due to the pandemic. The 1-2-3 Magic courses were run by the National Attention Deficit Disorder Information and Support Services (ADDISS), who are the sole license holders for 1-2-3 Magic in the United Kingdom. Initially, the trial protocol allowed for the recruitment of 84 participants who were successfully recruited from January 7 to February 10, 2022, which is 2 weeks earlier than the target date. The trial protocol was amended to allow for a total of 100 participants instead to maximize sample size and power, and the whole recruitment was completed in 9 weeks from the start date.

This study within a trial [19] aimed to evaluate the use of Facebook groups for participant recruitment for the web-based 1-2-3 Magic intervention program in parents or caregivers of children with ADHD in the United Kingdom.

### Social Media Platform: Facebook Groups

Facebook, a social media platform founded by Mark Zuckerberg in 2004, has been one of the most popular social media sites in the early 2000s [20]. Facebook has a “groups” feature where consumers are able to create and join any groups of their particular interests. Interests include but are not limited to sports, food, and science. There is a lot of freedom on Facebook to create any interest groups; for example, there is a group named “Students against backpacks with wheels” [21]. Facebook groups have brought communities together, and like-minded people have been provided with a space to network and interact. Many people use Facebook groups as online support groups [22,23]. This is a particularly helpful feature that researchers should use, especially if recruiting from a particular demographic. Thus, for this trial, we posted specifically in Facebook groups for parents of children with ADHD in the United Kingdom and UK ADHD support groups.

### The Difference Between Facebook Groups and Pages

The difference between a Facebook group and a Facebook page is that a Facebook page is designed to be run by an organization, business, or brand in order to market and promote its products and services [24]. However, Facebook pages are not limited to businesses, and any individual can create a page for any purpose. On the other hand, a Facebook group specifically aims to gather a community of people with the same interests or goals, such as ADHD parent support groups. Privacy settings between pages and groups may also differ. Pages are always open to the public to view, whereas some groups are private and require strict monitoring of activity by group admins, depending on the privacy settings that have been chosen by the creator of the group. Our trial used both groups and pages. We found it particularly helpful to partner with an organization that runs pages and groups catering to the demographic that we were recruiting. For example, ADDISS runs 2 pages (ADHD in Barnet, n=338 and ADDISS, n=4200) and 2 groups (ADHD Support group UK, n=10,878 and ADHD Awareness-ADDISS, n=1547). We posted twice in the ADHD Support group UK group on January 12 and March 4, 2022; once on the ADDISS page on January 7, 2022; once on the ADHD in Barnet page on March 2, 2022; and once on the ADHD Awareness-ADDISS group on February 4, 2022.

### ADDISS Facebook Groups and Pages

ADDISS was founded by AB (founder and chief executive officer) in 1997. They offer a wraparound service of support for families of children with ADHD and for individuals, whether young adults or adults with ADHD. The service offers information about ADHD and treatment routes; support and advice (one-to-one and group); and training for parents, health care professionals, and teachers. They also hold conferences and ADHD-related events [25]. ADDISS runs 2 Facebook groups and 2 Facebook pages.

### Digital Flyer and Facebook Post

**Figure 1:**
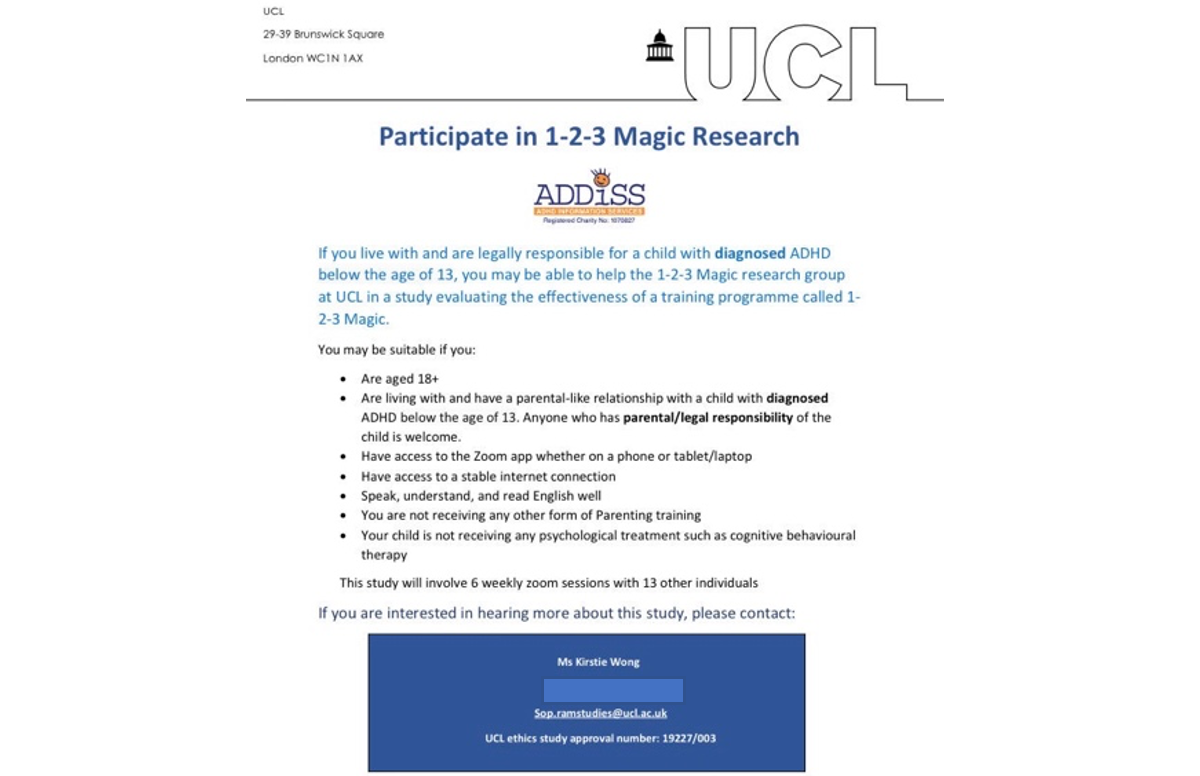
The digital recruitment flyer, disseminated on Facebook, for the recruitment of participants in the randomized controlled trial evaluating the effectiveness of 1-2-3 Magic delivered remotely for families in the United Kingdom of children diagnosed with attention-deficit/hyperactivity disorder (ADHD). UCL: University College London.

The design of the digital flyer (Figure 1) was created to catch attention, be concise, and be easy to read. We chose a white and blue color theme as the color blue is eye-catching, has been found to be calming, and increases alertness [26]. Research has also found that people associate the color blue with trustworthiness [27]. We made sure to clearly state the inclusion and exclusion criteria so as to limit the number of ineligible responses. We used bullet points to be concise and easy to understand.

**Figure 2:**
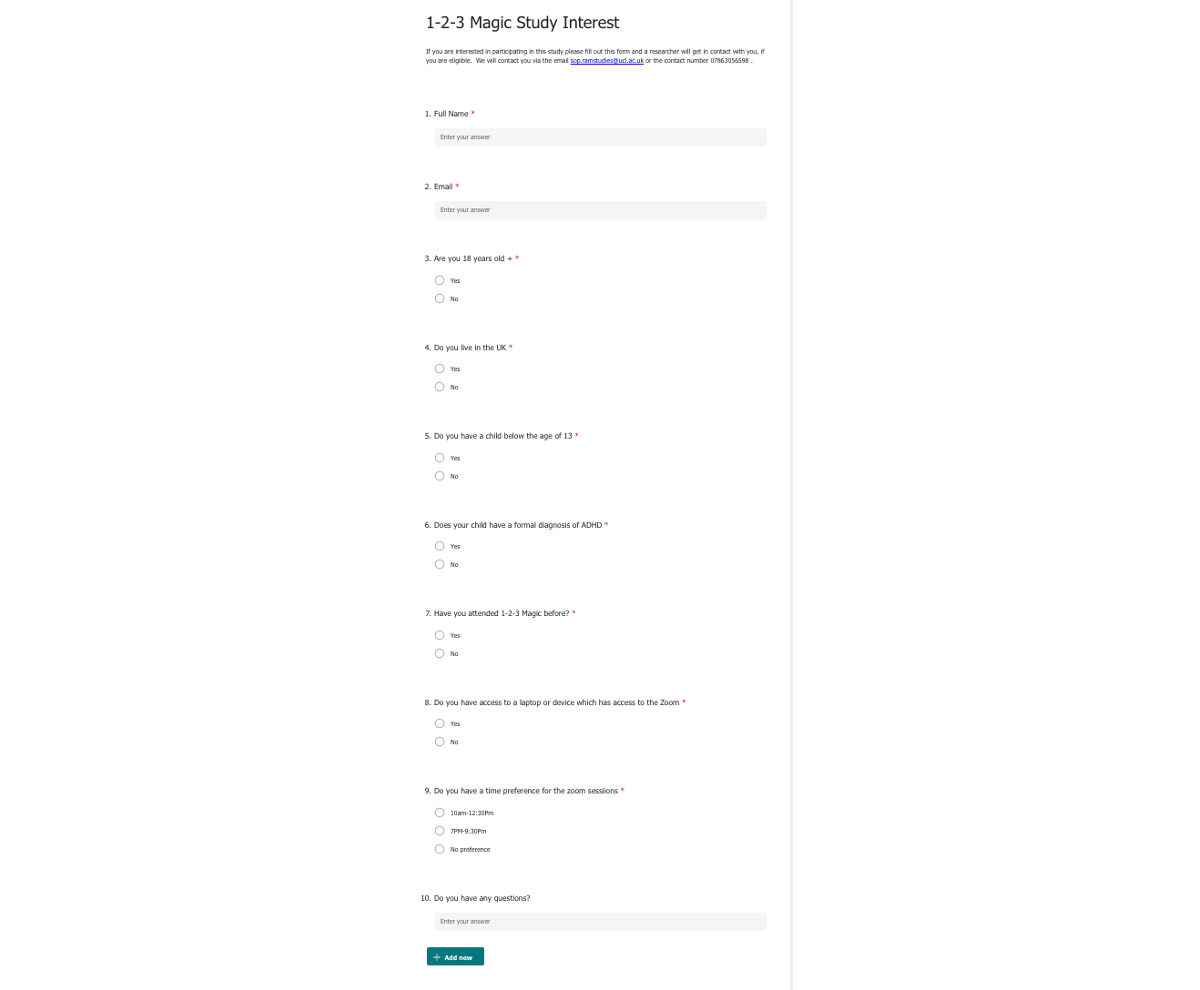
Microsoft Forms attached to the Facebook posts for prospective parents and caregivers to register their initial interest in the 1-2-3 Magic randomized controlled trial.

Facebook posts with a digital flyer with information about the 1-2-3 Magic trial were uploaded onto 2 ADHD Facebook groups and 2 pages at 5 different time points: January 7 and 12, February 4, and March 2 and 4, 2022. The groups had a combined total of around 1200 participants, and the pages had around 4200 followers. The flyer used is shown in Figure 1. The advertisement targeted parents of children aged 13 years or younger with a formal diagnosis of ADHD. A short synopsis of the trial was given, and the inclusion and exclusion criteria were stated, along with a contact number and email address. Alongside the flyer, a Microsoft Forms (Microsoft Corporation) link was attached so that parents who were interested could have a simple way of registering their interest. The form contained a total of 10 questions (Figure 2).

### Recruitment Procedure

**Figure 3:**
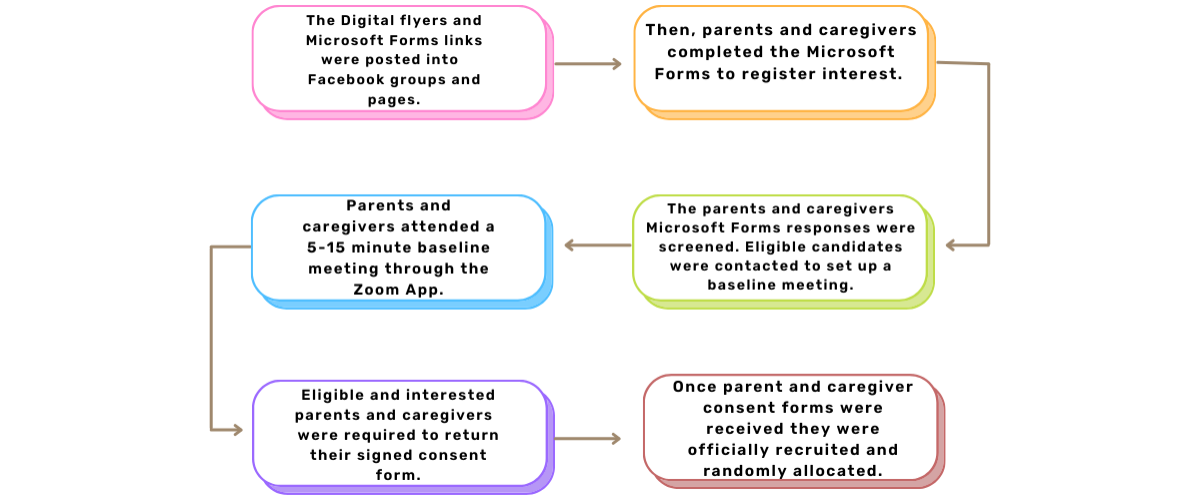
Recruitment flowchart outlining the methods used to recruit for the 1-2-3 Magic randomized controlled trial.

Once participants registered their interest through Microsoft Forms, they were emailed details to set up a baseline meeting using a website called Calendly [28]. Calendly allows the admin to set available time slots for participants to choose from. Once a meeting is set, the details of the Zoom (Zoom Video Communications) link for the meeting are emailed to the participants. A reminder email was also sent to participants on the day of the meeting, which contained the parent information sheet and the consent form. The baseline meeting typically only took 5 minutes. In the meeting, the researcher asked the parents to confirm their eligibility through a series of questions following a script. If at any point parents were not eligible for the trial, they were verbally made aware and asked if they would like to be put on a 1-2-3 Magic waitlist for courses independent of the trial. If parents were eligible, they were asked if they had read the information sheet and if they had any questions. They were asked if they understood what was required of them (attending the 6-week course, being randomly assigned to 1 of 3 time points, and completing 2 sets of surveys at 6 different time points). They were also made aware that they could withdraw at any time without any change of care from ADDISS or University College London. When the researcher had confirmed eligibility and parents were satisfied with the process, they were asked to return the signed consent form by email and complete the baseline set of surveys. Consent forms could be signed by pen and then scanned, or a picture of their signature or a digital signature could be used; typed names were not accepted. Figure 3 depicts the recruitment flowchart.

### Data Analysis

**Figure 4:**
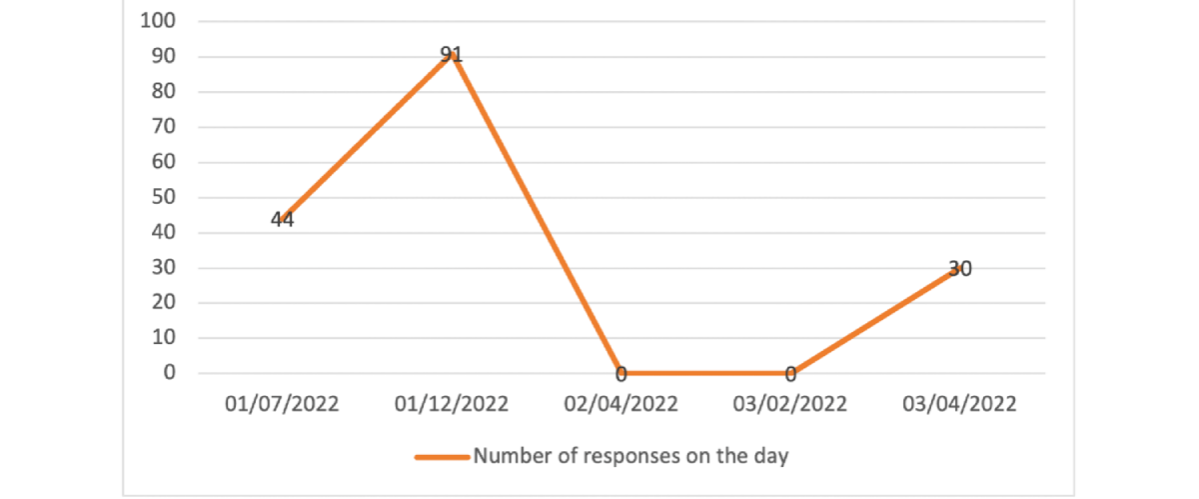
Number of participant responses on the day Facebook posts were disseminated for the 1-2-3 Magic randomized controlled trial.

Descriptive measures were used for this paper. Responses were counted to measure how many responses were generated on the day of a Facebook post being posted (Figure 4). Responses were measured by how many Microsoft Forms responses were submitted; for example, on the first post uploaded on January 7, 2022, a total of 51 Microsoft Forms responses were submitted. Second, we measured the overall number of Microsoft Forms responses received per week (Table 1). Third, we measured the number of people who reacted to each individual post (Table 2). Reaction to a post is defined as commenting, liking, or reacting using the available react buttons and sharing the post. Lastly, we evaluated the percentage of eligible responses in order to determine whether using this method of recruitment was successful in attracting the right demographic for our RCT.

**Table 1:** Number of responses per week.

Time period	Number of responses through Microsoft Forms (N=478), n (%)
January 7-14, 2022	208 (43.5)
January 15-22, 2022	129 (27)
January 23-30, 2022	27 (5.7)
January 31 to February 7, 2022	2 (0.4)
February 8-15, 2022	4 (0.8)
February 16-23, 2022	4 (0.8)
February 24 to March 3, 2022	0 (0)
March 4-11, 2022	94 (19.7)
March 12-19, 2022	4 (0.8)
March 20-29, 2022	6 (1.3)

**Table 2:** Facebook reactions per post.

Post date	Like, n	Comment, n	React, n	Share, n
January 7, 2022	6	13	0	14
January 12, 2022	14	21	1	0
February 4, 2022	0	0	0	0
March 2, 2022	2	2	0	1
March 4, 2022	9	26	4	0

### Ethical Considerations

Ethics approval for the recruitment of human participants for the 1-2-3 Magic RCT was obtained by the University College London Ethics Committee (19227/003). Written informed consent was given by all participants. All study data were anonymous and deidentified. Compensation of a £5 (US $6.07) Tesco voucher upon completion of the outcome surveys in the RCT was offered to the participants; however, this was not in the original protocol. The protocol was amended after the recruitment flyers had been distributed; thus, compensation was not an incentive for participants to participate in the RCT.

## Results

The number of Microsoft Forms responses is recorded in Figure 4 and Table 1.

Figure 4 displays the number of Microsoft Forms responses on the day of the published Facebook post. A total of 478 participants responded to the Facebook post by filling out the Microsoft Forms over a period of 2 months. After screening their responses, a total of 404 (84.5%) out of 478 parents were deemed potentially suitable for the RCT. All 404 parents were contacted to set up a baseline meeting. Out of the 404 parents, 135 (33.4%) responded and set up a baseline meeting; if they met the inclusion and exclusion criteria, they were enrolled in the trial. The first 100 participants to return their signed consent forms and complete the baseline surveys were officially recruited into the 1-2-3 Magic RCT.

The 5 Facebook posts were shared by a total of 15 individuals (Table 2). However, due to Facebook privacy settings, it is not clear how many more individuals shared the posts that had been shared. For example, the chief executive officer of ADDISS shared the Facebook advertisement, which led to another 27 shares, 21 likes, and 17 comments. Thus, the number of people the post would have reached is far greater than we can determine. Individuals who commented on the Facebook posts to register interest were directed to the Microsoft Forms link and were only emailed once they had filled out the Microsoft Forms. It is also important to note that 0 post interactions on a post does not equate to 0 Microsoft Forms sign-ups, as it was not a requirement to interact with the Facebook post to register interest for the RCT.

Respondents came from all over the United Kingdom, and those recruited in the trial predominantly resided in England (95/100, 95%). The other 5% (5/100) were from Scotland (3/100, 3%), Northern Ireland (1/100, 1%) and Wales (1/100, 1%).

We noticed that the post posted on March 2, 2022, generated 0 responses. This was perhaps because it had a small member list of 338 in comparison to the other groups, which had 10,900 and 1500 members. It was also possible that a lot of the parents were overlapping from other groups, so they had already registered their interest, as we had already posted in some of the largest UK ADHD support groups on Facebook.

Out of the 100 participants officially recruited, 92% (92/100) were Caucasian. The rest of the parents and caregivers were mixed race (3/100, 3%), East Asian (3/100, 3%), Arab or Middle Eastern (1/100, 1%), and South Asian (1/100, 1%). The lack of ethnic diversity could be due to cultural differences. Mental health is widely stigmatized in various cultures [29-32]. This may lead to parents and caregivers who are less inclined to accept outside help in relation to mental health [31]. Moreover, parenting styles differ between Western and non-Western cultures, which could also affect the likelihood of seeking parenting help [33]. Another factor to consider is that ADHD is more likely to be diagnosed in Western cultures than in non-Western cultures. Furthermore, people from non-white ethnic groups, particularly Asian, Black, and Hispanic individuals, are significantly less likely to be diagnosed with ADHD than their white counterparts [34,35]. This is not a criticism of the use of Facebook as a recruitment tool, but rather of the health systems that display racial disparity in mental health diagnosis.

Out of 478 responses, only 74 (15.5%) parents were not eligible. Due to Microsoft Forms, it was a simple and quick process to identify ineligible respondents. Ultimately, using Facebook was successful in magnetizing eligible participants, and the further use of Microsoft Forms enabled an efficient screening process with minimal human resources (a single researcher recruited all 100 participants).

## Discussion

### Overview

This study evaluated the effectiveness of recruitment for an RCT solely using established Facebook groups and pages. This method generated 478 responses through 5 Facebook posts spread out over a 2-month period. We successfully recruited our target sample of 84 participants 2 weeks ahead of schedule, allowing us to recruit an additional 16 participants. Recruitment for clinical trials is the most tedious part of the process and the most critical, as it is imperative to the success of the trial to recruit enough participants [36]. Previous papers published on the use of newspaper advertisements for recruitment have deemed them to be ineffective and potentially cost prohibitive [37]. Finding cost-effective and efficient distribution techniques to recruit for clinical trials has been explored in recent years [36]. However, this is the first published trial to have used established Facebook groups to recruit for an RCT. Through this method, we recruited 100 participants for an RCT in a short period of time. While advertisement campaigns, such as targeted Facebook advertisements, have been reported to be cost-effective, they still require funding.

### Previous Methods Used in Other Studies

Other studies have used Facebook using a variety of methods, such as targeted Facebook advertising [8,9], finding participants through the Facebook search bar [8], and creating Facebook pages [8]. Targeted Facebook advertising is the option to pay to post an advertisement on Facebook. Targeted Facebook advertising allows the user to choose targets and specific audiences based on characteristics including age, gender, marital status, and geographic region. Targeted Facebook advertisements are charged based on how many clicks the advertisement generates (cost per click); however, you can also set a budget. The cost per click can range from £0.76 (US $0.92) to £1.06 (US $1.29) [38]. Established Facebook groups and pages to recruit for RCTs remain unexplored.

In the meta-analysis [8], the authors found 1 study conducted in 2008 [39], which used the Facebook search bar to search for female adolescent participants involved in a previous study in order to conduct a follow-up study. However, this technique may create General Data Protection Regulation (GDPR) complications in the current day and age.

Lastly, a total of 2 other studies created their own Facebook pages to recruit participants [40,41]. A study [40] was only able to generate 127 responses across Facebook and Myspace (Time Inc). Most of their participants were recruited from face-to-face (30%) methods or referrals (59%), and only 3% were recruited from their Facebook and Myspace pages [40]. The second study was only able to recruit 6 participants through their group [41]. Our trial, in comparison, generated 478 responses from Facebook posts alone. It is important to note that both of those studies were qualitative or survey studies, and the majority of studies included in the meta-analysis, which used targeted Facebook advertisements, were also survey studies [8,12,16]. Only a few of the studies using targeted Facebook advertisements were RCTs and pilot studies [9,10,13,14]. Regardless, our method differs from these previously used methods. While a previous study created its own page, this is the first published trial that used already-existing databases of potential participants from Facebook groups and pages.

### Comparison With Previous Studies

There have been multiple studies over the years that have explored using Facebook to recruit for trials and studies. However, ours is substantially different for many reasons. First, our trial was more cost-effective. We did not pay to post about our trial, whereas the majority of studies and trials used targeted Facebook advertisements [8]. Second, our posts generated more responses compared with other papers, one of which used existing groups and pages [17] and 2 of which created their own Facebook pages [40,41]. Third, the recruiter for the 1-2-3 Magic trial did not have to actively seek out participants; interested participants were able to quickly and efficiently complete Microsoft Forms to register their interest. Other studies were less time effective as they were sending friend requests on Facebook to potential participants and thus had to wait for a response, explain to every individual what the trial was about, and wait again to see if the participant was either eligible or wanted to participate [40]. It is important to note that although 1 study [17] did use existing Facebook pages for their survey study, they deemed the method unsuitable for quantitative studies. However, their study was conducted in 2014, and due to the spike in internet traffic caused by COVID-19, Facebook groups may be better used nowadays. Despite their conclusions, our trial generated 478 results with 5 posts in 4 groups and pages; theirs generated 89 and 79 responses over 129 groups and pages. While their study was successful in sending surveys through a link in the advertisement, this is still the first trial known to use established Facebook groups and pages to recruit for a quantitative clinical trial. The experience from our trial suggested that this method was not just cost-efficient but also time and energy efficient. To conclude, our trial’s findings did not support the findings of the previous study.

### Pros and Cons of Using Facebook to Recruit Participants

Based on our experience using Facebook groups and pages to recruit participants in our RCT, we noted several strengths of this method of recruitment. The first strength is that it took much less time to complete the recruitment through Facebook groups than liaising with clinical services. We had set up a partnership with the London National Health Service’s (NHS’s) Child and Adolescent Mental Health Services (CAMHS) before recruiting participants; however, by the time they responded, we had already finished recruitment. This highlights the efficiency of direct recruitment through Facebook groups, as it does not rely on other parties.

The second strength is cost-effectiveness. The use of Facebook groups was completely free and did not involve any costs for publishing, stamps for letters, or phone call bills. Using Facebook groups, which already have hundreds of potential participants who already fit into your inclusion criteria, is also more time effective than advertising to the general public, which is costly and may not generate many relevant responses. A previous study that evaluated how effective newspaper advertising was for RCT recruitment generated 320 responses at a cost of £46,250 (US $56,151.43) [37], in comparison to our trial, which generated 478 responses in a 6-week time period and with no money spent. Due to the nature of their studies (surveys and questionnaires), it is unclear how effective paid advertising would be to recruit for a clinical trial, and further research should be conducted to compare the effectiveness of posting on Facebook groups versus paying for Facebook advertisements. For a larger study or trial, posts could be posted in more groups. As ours was a small trial, 2 groups and 2 pages generated more than enough interest.

A third strength is that, depending on the targeted sample size of the clinical trial and the topic of interest, finding participants through traditional methods may be tedious. For example, finding participants who have ultrarare conditions such as fibrodysplasia ossificans progressiva, where the prevalence is unknown but estimated to be 0.5 per million [42], may not be successful through typical recruitment methods. In contrast, there is a Facebook group for fibrodysplasia ossificans progressiva, which has 4300 followers. [43] Generally speaking, for a condition such as ADHD, which is one of the most common childhood neurological disorders [44], there are ample groups with thousands of members, and this is the case for most topics of interest. Thus, the decision to use Facebook groups for recruitment should take into consideration the topic being researched, and a quick Facebook search for groups and pages would be recommended beforehand.

There are also some limitations to using Facebook groups to recruit participants for clinical trials.

It may exclude individuals who experience digital poverty and do not have access to Facebook or individuals who do not use Facebook. While this approach has proven to be cost-effective and efficient in recruiting participants, it leaves out a segment of the population that is not on the Facebook platform and therefore may not be aware of the trial. Thus, it is important to acknowledge that this approach may still have inherent limitations in terms of inclusivity and representation for participants who face digital poverty and lack access to web-based platforms. However, to eliminate this bias, we did use other means of advertising in our trial, for example, word of mouth, LinkedIn, Twitter, Instagram, the ADDISS mailing list, and magazines. Overall, these methods only generated 4 email responses and 2 SMS text message responses. The majority of the responses were from Facebook, and ultimately, we recruited all of our participants from those who responded through a Microsoft Forms link in the Facebook group. This further highlights the superiority of using Facebook groups and pages for recruitment in this clinical trial. We did not recruit any parents through other means of advertising due to two reasons: (1) they did not fit the inclusion criteria, where 3 responders lived outside of the United Kingdom (India, Hong Kong, and Jersey) and others did not have a child who had been diagnosed with ADHD or did not fit other aspects of the eligibility criteria; or (2) they did not respond to booking a baseline meeting.

Research has suggested that people do not seek help to avoid learning about new information in order to maintain avoidant behaviors [45]. Thus, the second limitation to consider is that parents who have joined Facebook support groups and information pages may have a particular type of personality who may be more open-minded and willing to receive help, try new techniques, and learn new information. This could create a bias if all the participants have similar outlooks on life. However, in our trial, we found a wide range of optimists and pessimists and various expectations of the program beforehand. Many parents reported feeling skeptical about the course, while others reported feeling excited. Thus, among the 4200 individuals in the Facebook groups and pages used in this trial, it is reasonable to assume there are a wide range of personalities. However, we cannot assume other pages are the same.

A third limitation is that many Facebook groups do not allow individuals to post about research projects for safety reasons, and they risk being banned from the group if they do. This is something that we did encounter; however, partnering with ADDISS allowed us to post in more groups as they are already an established ADHD charity and allowed us to post in ADDISS groups and pages that have a substantial number of members and followers.

While a few studies have found newspaper advertising to be effective for recruitment [46], others have deemed it not effective [37]. Moreover, in this day and age, there has been an increasing tendency for people to use social media and internet rather than read physical newspapers, especially for public health information [47]. Society has been living in the digital age since the 1980s [48], but it is now more so than ever, heightened by COVID-19 [1]. Thus, the use of social media and Facebook groups in particular should be considered a superior method for recruitment for certain groups of participants.

### Strengths and Limitations of This Study

This study has notable strengths to consider. First, this is the first study, to our knowledge, after a literature review search, to consider the use of established Facebook pages and groups to recruit for a quantitative RCT, and we have found this novel method to enhance both economic and time efficiency. Second, the RCT in which we used this method to recruit is the first ever RCT to evaluate the web-based effectiveness of 1-2-3 Magic. Not only is the 1-2-3 Magic course valuable and educational, but the planning, recruiting, and successful execution, without delay, of the RCT during a global pandemic is a particularly novel experience that future research can learn from.

While this trial has unique strengths, it is important to note the limitations. The first limitation is that the data analysis was not as accurate as other methods could have been. For example, paid advertisements would be able to tell you how many people clicked on the advertisement and how many specific responses that one post generated. However, we did not have access to the exact number of Facebook posts the respondents had seen or the exact number of responses each individual Facebook post had generated. Therefore, this trial was only able to record the number of responses collected by date.

Second, others should interpret these findings with caution, as Facebook was a complementary method for this particular RCT; however, these findings may not apply to all other RCTs. For example, it is important to consider certain factors, such as the age of the target demographic, when deciding whether to use Facebook groups to recruit for a clinical trial. As parents with children aged 13 years or younger are generally in a younger age bracket, they are more likely to be tech-savvy and have access to social media and various consoles. This is reflected in our trial, as 80% (80/100) of the participants were aged between 25 and 44 years. Thus, if the target recruitment age is 50 years or older, it may be better to consider a different recruitment method.

### Conclusion

In conclusion, we found that digital flyers posted in the targeted Facebook groups to recruit participants for a clinical trial, which opened during the COVID-19 pandemic, were a successful tool to enhance the efficiency and cost-effectiveness of recruitment. The primary implication of these findings is that, irrespective of the presence of an ongoing global pandemic, trialists should consider this low-cost recruitment method. This novel recruitment method, which enabled the trial to persist successfully during a global pandemic when many others failed, underscores the potential value of harnessing social media for research study recruitment.

## Abbreviations

ADDISS: National Attention Deficit Disorder Information and Support Services
ADHD: attention-deficit/hyperactivity disorder
CAMHS: Child and Adolescent Mental Health Service
GDPR: General Data Protection Regulation
NHS: National Health Service
RCT: randomized controlled trial

## Data Availability

Data sharing is not applicable to this article as no data sets were generated or analyzed during this study within a trial.
